# The Present Scarcity of Nurses

**Published:** 1920-05-29

**Authors:** 


### THE MATRONS' AND SISTERS' DEPARTMENT.

### THE PRESENT SCARCITY OF NURSES.

It is rumoured that the Medical Consultative Council has recommended the Minister of Health to throw the onus of providing nurses for the sick on the local authorities. If this comes to pass, the unhappy municipal authorities will be compelled by law to provide a nurse for every patient who is certified by a doctor to require one. Thus it is believed the wards of the hospitals will be relieved and home nursing be indefinitely expanded.

The bare suggestion of making it a legal obligation for the Mayor and Corporation to find nurses for all the sick persons in the city brings into relief the acute shortage of nurses under which the community groans at the moment. This shortage is felt in every single department of the health services. To start with, there are not nearly sufficient candidates for training. There are not enough charge or staff nurses for institutions which cannot train their own. There is grave difficulty in obtaining ward sisters. Private nurses are needed in every direction. Most nursing homes are embarrassed for lack of nurses. As for district and village nurses, they are almost as scarce as houses. It would be as reasonable to expect the local authorities to find houses at once for all who are looking for them as to expect them to find nurses for all who are sick. Are we to conclude from the scarcity of nurses that women are abjuring the profession of nursing, just as it is becoming a career with good prospects?

Before reaching such a sensational conclusion it may be well to examine the facts. During the years of war the profession was depleted to the extent of from 20,000 to 30,000 nurses, all in their prime. We shall probably never be able to form any judgment as to the number of war nurses who abandoned their profession at the end of their service, but it undoubtedly reaches a very high proportion. A large number married either during or after the war. A large number relinquished nursing from reasons of disablement, small or great. Of the rest, comparatively few were available for filling up the ranks of general utility nurses. Their work had been too highly specialised. Their responsibilities had raised them above the status they would have occupied had nothing interrupted the normal routine of their career. Thus, even if there were no other cause, a shortage of nurses could not fail to be experienced at the present moment as a consequence of the war.

But with all this dislocation, the number of nurses has demonstrably increased to an extent which is strangely ignored. The movement for affording nurses a forty-eight hours week and one day in seven, although it has by no means universally spread, has entailed in itself an increase of quite ten per cent. of nurses all over the kingdom. Poor-Law infirmaries formerly averaging twenty-five patients to each nurse are approaching by degrees an average of six to one. Almost every one of the major hospitals, whether or no they have introduced the eight-hours day, have added fresh departments which entail an increased staff, and every single one has made concessions about off-duty time to the staff. All this means that more and yet more nurses have to be employed. When we complain of the shortage shall we not at the same time be mindful of the enormously increased demand?

It is a demonstrable fact that never in the history of trained nursing have there been so many nurses in training as at the present time. The training capacities of the schools have been indefinitely expanded, as may easily be seen by the universal demand for larger Nurses' Homes. We are not to judge the desire of women to become nurses by the inflated statistics of some years ago published by matrons embarrassed by crowds of unsuitable applicants. There are many more training-schools now, and each one receives a number of probationers largely in excess of its former maximum. It is not that the harvest of good candidates is failing. It is that the number of those who cast the net to secure them has increased momentarily beyond the normal increase in the catch. In the days when the institutions giving training which could rank as "recognisable" were to be counted by tens, candidates were irresistibly drawn to these centres from all over the country. Now when, speaking roughly, they may be counted by hundreds, things are very different.

We are passing through a transition time, and patience is indicated. That is the long and the short of it. Believing as we do that there is no finer career for a woman than nursing, we rejoice at every sign that the community is determined to have more nurses, is now reconciled to the necessity for paying them well, and is beginning at last to understand the difference between good training and bad.

